# Emerging concepts in post-operative pain management

**DOI:** 10.4103/0019-5049.79872

**Published:** 2011

**Authors:** SS Harsoor

**Affiliations:** No. 21, 2^nd^ Cross, Kirloskar Colony, Basaveshwarnagar, II Stage, Bangalore - 560 079, Karnataka, India. E-mail: harsoorss@hotmail.com

Pain is a subjective and multidimensional experience, which is most often ignored by health care providers. Studies have found that the prior identification of patients at risk of experiencing post-operative pain will allow early intervention and better pain management. The current practice of using post-operative analgesics with a focus on patients’ demands may not be adequate. Drugs such as IV paracetamol, IV diclofenac, COX 2 inhibitors and opioids may not be meeting all the requirements of post-surgical patients.

Adequate post-operative pain relief must be an integral part of administration of anaesthesia. Untreated surgical pain may result in a decrease in alveolar ventilation and vital capacity and even pneumonic consolidation. It can cause tachycardia, hypertension, myocardial infarction, insomnia and poor wound healing. Importantly, post-operative pain management is included as one of the important discharge criteria in day care anaesthesia.

Inadequate post-operative pain relief may result in clinical and psychological changes that may increase the morbidity and mortality as well as the cost of treatment as a whole, in addition to decreasing the quality of life post-operatively. It may be associated with deep vein thrombosis (DVT), and pulmonary embolism, pneumonia, delayed wound healing and demoralization.[1] Realizing the problems of unrelieved acute pain, Joint Commission on Accreditation of Healthcare Organizations (JCAHO) has recommended standards of pain management, especially with regard to assessment, monitoring and treatment.

It is observed that up to 50% of patients may develop chronic post-operative pain including minor depression[2] and pain-related catastrophizing.[3] Hence a distinct element of chronic post-surgical pain (CPSP) is described, which is related to bio-psychosocial factors.[4,5] The depression, psychological vulnerability stress and late return to work are closely related to CPSP. This CPSP is not only observed following a major surgery but can be seen after a minor procedure like hernia repair or vasectomy.

The goals of preemptive analgesia include decreasing the pain after surgical injury, avoiding the pain-related pathological modulation of central nervous system (CNS) and preventing the persistence of post-operative pain.

A number of clinical studies have failed to demonstrate the claimed benefits of preemptive analgesia. It is now emerging that neuronal hypersensitivity and nociception after surgical incision are mainly maintained by afferent barrage of sensitized nociceptors across the post-operative period, quite contrasting to other types of injury.[6] Thus it is suggested that both analgesia and anti-hyperalgesic intervention are necessary in a post-surgical setup.

Equally important is the need to educate the patients about the communication of unrelieved pain. The practice guidelines issued by ASA Task force on Pain Management (1995) regarding acute pain have helped in promoting the standardization of techniques, equipments and drugs to a limited extent only. Several studies conducted to assess the impact of these guidelines have shown a very poor response.

The increase in the incidence of day care procedures in the recent years has thrown newer challenges for post-operative pain management. In a most cited article,[1] nearly 80% of patients experienced pain after surgery which was inadequately treated. Also, it was observed that proper education and treatment of post-operative pain has increased the positive psychological impact on these patients. In the past few years, we have seen major technological breakthroughs in the field of post-operative analgesia, which have greatly increased patient care and satisfaction also.

Inadequately treated post-operative pain can cause exacerbation of acute nociceptive pain resulting in allodynia and hyperalgesia. In a patient with primary hyperalgesia, peripheral nociceptors are sensitized, whereas in secondary hyperalgesia, there is sensitization at the spinal cord and CNS. During peripheral sensitization following primary hyperalgesia, there will be a release of primary mediators such as PGE, 5-HT, leukotrines, bradykinin, etc., which finally stimulate the release of substance P. These pain impulses from peripheral nociceptors travel via A-δ and C-fibers to Lamina II and Lamina V of the spinal cord. The C-fibers also synapse in Lamina I of the spinal cord. The neurons in Lamina V respond to both noxious and non-noxious stimuli through neurotransmitters such as glutamate and aspartate.[7]

The NMDA receptors need aspartate and glutamate ligands to regulate the flow of both Na+ and Ca+ and also K+ outflow. The entry of Ca+ ions is initiated by the removal of the Mg+ plug in receptors. At NMDA receptors, the entry and accumulation of Ca+ into neurons leads to rapid and independent firing of neurons even without any stimulation. Such activation of NMDA receptors in the spinal cord as well as supraspinal regions of CNS can ultimately result in the long-term potentiation (LTP) of pain. This central sensitization, which causes LTP is a reversible process and hence amenable to treatment (transcription independent process of sensitization).[7] But transcription independent sensitization is mediated by an alteration in the dorsal root ganglion and dorsal horn and can produce irreversible structured modifications in the CNS.

Hence the knowledge of the mechanism of both acute and long-term chronic pain has led to the concept of multimodal analgesia. The technological developments with regard to drugs and also the route of administration of these drugs have modified the way the healthcare providers were able to effectively treat and manage post-surgical pain.

## NEWER DEVELOPMENTS

### Capsaicin

The TRPV-1 agonist is a non-narcotic alkaloid acting peripherally at unmyelinated C-fiber nerve endings.[8] The presence of capsaicin causes a sustained release and ultimately exhaustion of substance P at nerve endings. However, it has minimal effects on A-δ fibers and does not affect temperature and touch sensations. When used as a cream along with narcotic and non-narcotic analgesics, it is thought to exhibit opioid-sparing effects. The injectable capsaicin is useful both for post-surgical pain and chronic long-term pain management. It is a safe drug but should be used with caution in patients with elevated liver enzymes and patients on ACE inhibitors.

### Gabapentin and pregabalin

Gabapentin is an antiepileptic drug which binds to the α-2 subunit of presynaptic voltage-gated calcium channels of GABA receptors and it prevents the entry of Ca+ into neurons, thus preventing the release of neurotransmitters responsible for the activation of the pain pathway. Oral gabapentin, 300-1200 mg, administration is known to produce an opioid-sparing effect during 24 h of the post-surgical period and is also known to have an anti-hyperalgesic property.

A derivative of gabapentin and structural analogue of GABA, pregabalin is known to produce analgesic, sedative and anti-anxiety properties. The reports about its role in acute pain management are equivocal, but its use in chronic pain is well established.[9] The properties such as the absence of respiratory depression, gastric-sparing effects and anxiolysis make pregabalin a useful adjuvant even in the management of post-surgical pain, but probably larger studies may be needed before the drug is advocated for routine use.

### Dexmedetomidine and clonidine

Selective α-2 agonist drugs like dexmedetomidine, 0.5-2 mg/kg IV, are known to have excellent anaesthetic-sparing and opioid-sparing properties, by unknown mechanisms.

### Local anaesthetics

The formulation of liposome- or polymer-encapsulated local anaesthetics can result in the prolongation of the duration of action.[10] Liposomes are non-immunogenic, and form a phospholipid layer, which are biodegradable and hence can prolong the drug release.

### Tapentadol

It is a new receptor agonist with 18 times more affinity than morphine and it also inhibits the noradrenaline uptake.[11] Still it is two to three times less potent than morphine, and the total oral maximum dose is 600-700 mg to be administered every 4–6 h in divided doses. The drug has better GI tolerance and can be used in patients with renal impairment also. The disadvantages include the possibility of hypertension, serotonin syndrome (hallucinations and autonomic instability) and hence it should not be combined with serotonin uptake inhibitors, noradrenalin uptake inhibitors or tricyclic anti-depressants. Tapentadol is contraindicated in patients with bronchial asthma, paralytic ileus, etc.

Extended release epidural morphine can provide a long-lasting pain relief up to 48 h without producing a higher systemic drug concentration.[12] Depodur (Pacira Pharmaceuticals, San Diego, CA) administered epidurally is found to provide long-lasting effects in total knee replacements and also in Caesarian section studies. However, it has failed to address the problem of opioid side effects such as pruritis and respiratory depression in elderly patients and hence close monitoring is essential.

### Fentanyl

The development of iontophoretic transdermal system (ITS) is a needle-free, pre-programmed drug delivery system which works by the application of low-intensity electrical fields across skin. This technology is yet to be approved and put into clinical practice.[13] It is observed that about 40% of the administered drug is absorbed in the first hour and the complete absorption and action may last up to 4 days. The adverse effects of fentanyl ITS were similar to those following IV administration and IV morphine. The other adverse effects such as skin hypersensitivity and long-term hyperpigmentation can be troublesome. Since the system lacks programmability, the patient education is very important.

### Adjuvant revisited

With the better understanding of the role of NMDA receptors in pain modulation, the use of a subanaesthetic dose of ketamine (0.5-1 mg) is finding a place in post-operative pain management, especially in preventing opioid-induced hyperalgesia. However, the possibility of side effects like hallucinations and blurred vision should be kept in mind.

### PCRA

Placing an indwelling perineural catheter with the help of a nerve stimulator/locator and ultrasound imaging is very effective, gratifying, and safe method to provide post-operative analgesia. Often the drugs like bupivacaine or ropivacaine are administered as infusion with preprogrammed elastomeric or electronic pumps. Even the use of adjuvants along with these local anaesthetics is reported with better safety and good results. The indwelling catheter can be placed in the surgical wound area, intra-articular or even intra-pleural locations to obtain the maximum benefit with minimal side effects and with only very low drug concentrations.

## FUTURE

Procedure-specific, evidence-based, acute post-operative pain management guidelines, based on patients co-morbid conditions, and psychological status may become the routine practice.
